# Immune Checkpoint Inhibitor‐Induced Endocrine Dysfunction: Early Detection of Symptoms and Timely Intervention

**DOI:** 10.1002/ccr3.72528

**Published:** 2026-04-14

**Authors:** Nadia Grace Obaed, Kadiyatu Fofana, Ahmad Matarneh, Sundus Sardar, Naman Trivedi, Nasrollah Ghahramani

**Affiliations:** ^1^ Department of Internal Medicine‐Pediatrics Penn State Milton S. Hershey Medical Center Hershey USA; ^2^ Department of Medicine Penn State Milton S. Hershey Medical Center Hershey USA; ^3^ Department of Nephrology Penn State Milton S. Hershey Medical Center Hershey USA

**Keywords:** adrenal insufficiency, endocrine toxicity, hypophysitis, immune checkpoint inhibitors, immune‐related adverse events

## Abstract

Immune checkpoint inhibitors (ICI) target immune regulatory pathways to enhance antitumor responses. Although these agents improve survival, they can cause immune‐related adverse events (irAEs). Hypophysitis is a recognized irAE—particularly with CTLA‐4 inhibitors—but this case is notable for occurring in an elderly patient receiving combination therapy and presenting with nonspecific symptoms that mimicked cancer‐related fatigue and treatment side effects. We report a 76‐year‐old woman on ICI therapy who developed intractable back pain, nausea, and severe hyponatremia, ultimately diagnosed with ICI‐induced hypophysitis. Her evaluation revealed multiple electrolyte abnormalities, secondary adrenal insufficiency, and pituitary enlargement on magnetic resonance imaging (MRI), necessitating urgent fluid resuscitation and corticosteroid replacement. As use of ICIs expands across malignancies, understanding the side effect profile and atypical or subtle presentations is essential in providing prompt diagnosis and treatment of sequelae that can mimic autoimmune diseases. It underscores diagnostic complexity in real‐world settings and the need for heightened awareness of adverse endocrine events, especially in older patients and those receiving sequential or combination regimens. Careful monitoring of irAEs allows for irAEs risk mitigation and improves patient tolerability in continuing ICI cancer treatment. Clinicians should maintain a high index of suspicion and implement appropriate monitoring protocols for early detection.

## Key Clinical Message

Combination ICI therapy accelerates the onset of hypophysitis compared to monotherapy, with symptoms emerging within weeks rather than months. Clinicians should maintain heightened vigilance for nonspecific symptoms such as fatigue and hyponatremia in patients transitioning to combination regimens to enable early glucocorticoid intervention.

## Graphical abstract

Immune checkpoint inhibitors (ICIs) can cause immune‐related adverse events (irAEs), including hypophysitis—particularly with CTLA‐4 inhibitors. Atypical presentations in older patients may mimic cancer‐related fatigue or treatment side effects, delaying diagnosis. A 76‐year‐old woman on combination ICI therapy presented with intractable back pain, nausea, and severe hyponatremia. Workup revealed secondary adrenal insufficiency and pituitary enlargement on MRI, consistent with ICI‐induced hypophysitis. She required urgent fluid resuscitation and corticosteroid replacement. Nonspecific symptoms (fatigue, back pain, nausea) masked underlying hypophysitis. Laboratory evaluation showed multiple electrolyte abnormalities and secondary adrenal insufficiency. MRI demonstrated pituitary enlargement. Prompt corticosteroid replacement enabled clinical recovery. Clinical Message: As ICI use expands, clinicians should maintain a high index of suspicion for endocrine irAEs—especially in elderly patients and those on combination regimens. Early recognition and appropriate monitoring allow timely treatment and support continued ICI therapy.

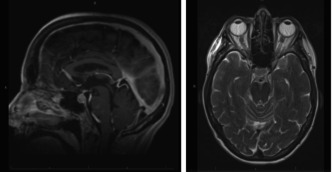

## 1 Introduction

Immune checkpoint inhibitors (ICIs) have transformed cancer therapy by activating antitumor immunity but also lead to immune‐related adverse events (irAEs), including damage to healthy tissues. Among the various irAEs, endocrine disorders like hypophysitis are notable. Hypophysitis, characterized by inflammation and dysfunction of the pituitary gland, is most frequently associated with cytotoxic T‐lymphocyte‐associated protein 4 (CTLA‐4) inhibitors such as ipilimumab and occurs more often with combination regimens involving blockade of programmed cell death protein 1 or programmed death‐ligand 1 (PD‐1/PD‐L1) [1, 2]. The presentation of ICI‐induced hypophysitis is often insidious and nonspecific, including symptoms such as fatigue, headache, and nausea which overlap with malignancy or treatment‐related symptoms [1, 2].

Diagnostic complexity increases in older adults and patients on sequential or combination therapy. Diagnosis typically involves measuring anterior pituitary hormones and thyroid function. Thyroid dysfunction, often presenting as transient thyrotoxicosis followed by hypothyroidism from destructive thyroiditis, is another common ICI‐related endocrinopathy [3].

Clinically, patients classically manifest symptoms within 6–12 months after initiating ICI therapy [2]. This case highlights the diagnostic complexity of ICI‐induced hypophysitis, particularly in elderly patients with subtle or atypical symptoms. Additionally, less is known about how sequential or combination therapy modifies presentation and clinical course. Early identification and prompt management, including hormone replacement and supportive care—are essential to prevent life‐threatening complications such as adrenal crisis and to allow continuation of oncologic therapy. As the use of ICIs continues to expand, clinical vigilance and systematic endocrine monitoring are critical to minimizing morbidity and optimizing chemotherapy outcomes.

## 2 Case Presentation

A 76‐year‐old female with angiosarcoma of her bilateral breasts on maintenance nivolumab and ipilimumab status‐post Cycle 2, and history of mucinous right breast cancer status‐post radiation therapy and papillary thyroid cancer status‐post thyroidectomy on 100 mcg levothyroxine was admitted for intractable back pain and severe hyponatremia. She had transitioned to combination therapy 1 month after nivolumab monotherapy because of disease progression. She reported acute worsening of chronic back pain at a known T6 fracture, fatigue, nausea, poor appetite, and bilateral breast pruritus.

## 3 Diagnostic Assessment

Initial labs showed sodium at 121 mmol/L and significantly decreased TSH (0.01 uIU/mL). On presentation, her vitals were stable and the exam showed breast inflammation and thoracic spine tenderness without neurologic deficits.

**FIGURE 1:**
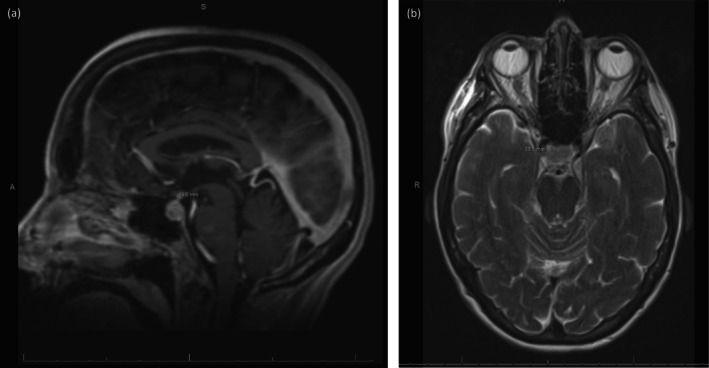
MRI Showing Pituitary Enlargement with Differential Considerations. (a) A sagittal T1‐weighted MRI image of the brain demonstrates that the pituitary gland is enlarged for age, with a convex upper border and measuring approximately 13 mm. (b) An axial T2‐weighted MRI image of the brain demonstrating hypodensity and enlargement of the pituitary gland approximately 13.1 mm in diameter. The differential diagnosis of this image alone includes adenoma, hypophysitis (associated with some immune checkpoint inhibitor), dysplasia, and metastatic disease.

She was initially fluid restricted for presumed euvolemic hyponatremia. Endocrinology was consulted and an MRI brain was ordered with an endocrine panel (thyroid‐stimulating hormone (TSH), T3, T4, morning cortisol, adrenocorticotropic hormone (ACTH), dehydroepiandrosterone sulfate (DHEAS), urine electrolytes). Results showed low TSH (0.01 uIU/mL), free T3 (1.5 pg/mL), cortisol (2.4 ug/dL), ACTH (< 1 pg/mL), and DHEAS (30 ng/mL). Urine urea was 304 mg/dL and urine creatinine was 60 mg/dL. MRI brain demonstrated significant pituitary enlargement and intracranial lesions suspicious of metastatic disease (Figure 1A,B).

Given her pituitary enlargement, low cortisol and ACTH, and hyponatremia, an endocrinologist suspected ICI‐induced hypophysitis with secondary adrenal insufficiency. Furthermore, pituitary metastasis was an important differential diagnosis. There was also concern for cerebral salt wasting versus syndrome of inappropriate antidiuretic hormone (SIADH). A cosyntropin test was deferred to allow immediate treatment. Fractional excretion of sodium was under 1% and the fractional excretion of urea was under 35%, suggesting intravascular depletion.

## 4 Treatment, Outcome and Follow‐Up

**FIGURE 2:**
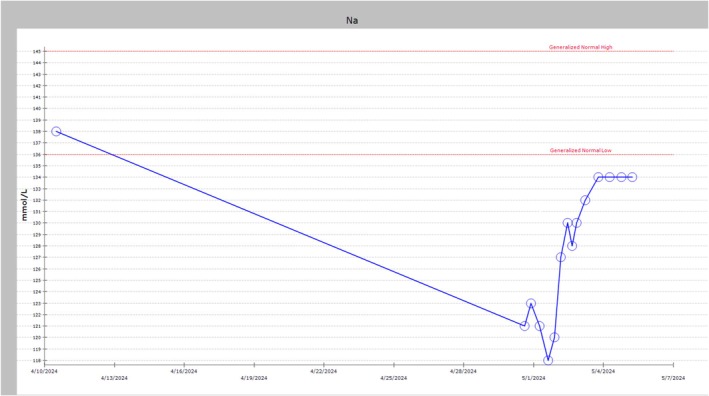
Serum sodium trend in mmol/L. The patient's Na prior to ICI‐induced hypophysitis was 138 mmol/L. Her sodium level decreased to 121 mmol/L 20 days later, prompting her hospital admission. Her Na level was frequently monitored and improved throughout her hospitalization.

Hydrocortisone 25 mg intravenous (IV) three times daily and maintenance IV normal saline were initiated. Her sodium briskly improved (Figure 2). While standard stress dosing for adrenal crisis is 100 mg IV bolus followed by 200 mg/day, a lower dose was selected given the patient's hemodynamic stability on presentation. The rapid improvement in sodium levels supported the adequacy of this approach in this clinical context. Hydrocortisone was tapered to 5 mg oral daily on discharge and continued indefinitely. Standard physiologic replacement for secondary adrenal insufficiency is 15–25 mg hydrocortisone daily, typically divided into two or three doses (e.g., 10–15 mg on waking and 5 mg in early afternoon) to approximate the circadian cortisol rhythm. Given the patient's transition to hospice care and limited life expectancy, a simplified once‐daily regimen was selected to minimize treatment burden, acknowledging that this approach prioritized comfort over strict physiologic replacement. Palliative care guided goals‐of‐care discussions, and the patient ultimately transitioned to hospice and passed away within a month.

## 5 Discussion

As ICIs become increasingly common, early recognition of endocrine irAEs, particularly hypophysitis, is essential to mitigate morbidity and support safe treatment continuation [4]. Epidemiologic data confirm associations between ipilimumab, nivolumab, and pembrolizumab with hypophysitis, with the highest reporting odds ratio for ipilimumab [5]. Although data on the diagnosis, natural course and management of ICI‐related hypophysitis remain limited [6]. Symptoms are often subtle and easily misinterpreted [7], as demonstrated by our patient. The American Society of Clinical Oncology and the American Association of Clinical Endocrinology recommend routine hormonal surveillance during the first 12 weeks of therapy, particularly in combination therapy [8].

Hypophysitis associated with PD‐1 or PD‐L1 inhibitors represents a clinically distinct and particularly insidious entity compared to CTLA‐4 inhibitor‐induced hypophysitis. While anti‐CTLA‐4 agents typically cause pituitary enlargement on MRI in up to 98% of cases, PD‐1/PD‐L1 inhibitor‐induced hypophysitis shows normal pituitary imaging in approximately half of patients and predominantly manifests as isolated ACTH deficiency [9]. The time to onset is considerably delayed with PD‐1 inhibitors, occurring at a median of approximately 26 weeks compared with 9 weeks with ipilimumab [9]. The median duration from symptom onset to confirmed diagnosis can be as long as 5.5 months, reflecting the nonspecific nature of symptoms such as fatigue and anorexia [10]. Additionally, PD‐1 blockade can trigger sequential or overlapping endocrinopathies that evolve over time; Rossi et al. described a case of pembrolizumab‐induced triple endocrine dysfunction involving thyroiditis, hypophysitis, and adrenalitis, in which central adrenal insufficiency developed after initial thyroid dysfunction and was subsequently followed by primary adrenal insufficiency [11]. Our patient's case, in which symptoms emerged after transitioning from nivolumab monotherapy to combination therapy, highlights the compounding diagnostic complexity when both PD‐1 and CTLA‐4 pathways are simultaneously targeted. Hyponatremia typically reflects secondary adrenal insufficiency due to reduced ACTH and cortisol levels, impairing sodium balance. MRI commonly reveals pituitary enlargement in the acute phase, which may later evolve to atrophy [12]. A notable MRI finding is geographic hypo‐enhancing lesions within the anterior lobe of the pituitary, consistent with prior reports [12]. These lesions often correspond to fibrosis and exhibit hypodensity on T‐2 weighted images as seen in Figure 1b suggesting a chronic inflammatory process. It is important to note that imaging is not universally diagnostic; absence of classic findings does not exclude hypophysitis [8].

Pituitary metastasis represented a critical differential diagnosis in this patient given her advanced angiosarcoma and the identification of intracranial lesions on imaging. Differentiating ICI‐induced hypophysitis from metastatic involvement of the pituitary poses a well‐recognized diagnostic challenge, as new pituitary abnormalities occur in up to 18% of cancer patients receiving ICIs [10]. Several clinical and radiologic characteristics supported hypophysitis over metastasis in our case. Metastatic deposits to the pituitary characteristically affect the posterior lobe and infundibulum first, frequently causing diabetes insipidus as an early manifestation [13]. Our patient had no clinical or biochemical evidence of diabetes insipidus, which is notably uncommon in isolated ICI‐induced hypophysitis; its presence should raise concern for metastatic disease [13]. Radiologically, ICI‐induced hypophysitis typically produces diffuse, modest, and homogeneous pituitary enlargement, whereas metastases tend to appear as discrete lesions with heterogeneous enhancement, extension into the cavernous sinus, sellar floor erosion, or clival invasion [14]. Our patient's imaging demonstrated diffuse pituitary enlargement with geographic hypo‐enhancing regions in the anterior lobe—a pattern characteristic of ICI‐induced inflammation and fibrosis rather than metastatic infiltration [14]. The biochemical profile showing deficiencies in ACTH, cortisol, and TSH with intact posterior pituitary function aligned with anterior hypophysitis rather than metastatic destruction [12]. The temporal association between combination ICI initiation and symptom development, combined with prompt clinical and biochemical improvement following glucocorticoid replacement, further supported an immune‐mediated process. Although pituitary biopsy remains the definitive method for excluding metastasis, a presumptive diagnosis is often achievable without tissue sampling when clinical, biochemical, and imaging findings are concordant [8].

In our patient, prompt initiation of steroids, and fluid management effectively addressed her acute symptoms and electrolyte imbalances, restoring normal sodium levels. Treatment centers on physiologic glucocorticoid replacement, with stress dosing for acute illness [8]. High‐dose steroids do not improve pituitary recovery, except in cases of symptomatic mass effect [8]. Recovery of corticotroph function is rare, necessitating lifelong glucocorticoid replacement in most patients, though approximately half may regain hypothalamic‐pituitary‐thyroid axis activity [8].

Combination ICI therapy increases the risk of endocrine toxicity. Trials including CheckMate 067 report hypophysitis rates up to 10%–15% with earlier symptom onset in combination ICI therapy, particularly regimens including CTLA‐4 inhibition [2]. Similarly, the incidence of hypothyroidism is higher with combination ICI therapy (13.2%) compared to PD‐L1 inhibitors alone (7%) [3]. Despite this, the accelerated timeline of endocrine irAEs, specific presentation, and diagnostic nuances following transition from monotherapy to combination therapy are underreported.

**FIGURE 3:**
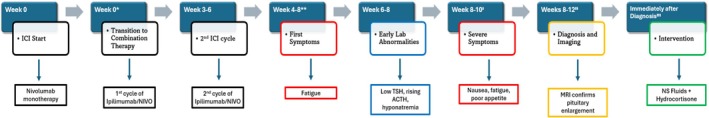
Timeline‐based progression model for ICI‐induced hypophysitis with patient case comparison. The top row demonstrates a timeline‐based progression model for ICI‐induced hypophysitis based on existing clinical guidelines to recognize and manage irAEs, published case reports, and cohort studies describing symptom onset, and the pathophysiology of ICI‐induced hypophysitis. Week 0 depicts initiation of immune checkpoint inhibitor therapy, which should be preceded by baseline pituitary function tests. Typically, routine monitoring during treatment occurs every 2–4 weeks, especially for combination therapy like nivolumab + ipilimumab. The bottom row depicts our patient's timeline. Both timelines track ICI administration, symptom onset (fatigue, nausea, etc.), lab abnormalities (TSH, ACTH, sodium, cortisol), imaging findings, and intervention. *Timeline accelerates now on combination therapy. **Combination therapy accelerates symptom onset compared to monotherapy, which may take up to 12 weeks. ꭞSevere symptoms may occur closer to Weeks 10–12 in monotherapy. ꭞꭞMay even shift closer to Week 6–8 in high‐risk patients such as older adults, patients with the presence of preexisting autoimmune conditions, and patients receiving high‐dose CTLA‐4 inhibitors. ꭞꭞꭞTypically, Week 8–12 for combination versus Week 10–14 for monotherapy.

In this case, symptoms emerged within weeks of initiating combination therapy. Her symptoms can be attributed to deficiencies in the anterior pituitary hormones, particularly ACTH. The case illustrates how the biochemical and clinical timeline is compressed in patients receiving combination ICI therapy, with earlier onset of symptoms (Weeks 4–8) than monotherapy (Weeks 8–12) (Figure 3). The accelerated timeline is particularly relevant for older patients who may be at higher risk due to reduced pituitary reserve and increased susceptibility to immune‐mediated pituitary inflammation [15]. Clinicians should maintain a high index of suspicion for irAEs in patients transitioning to combination ICIs. Robust monitoring protocols are critical to ensuring optimal outcomes for patients receiving combination ICI therapy.

As this represents a single case, the findings cannot be generalized broadly across all patients receiving ICIs. Variability in patient presentations, underlying comorbidities, and treatment regimens can influence management of irAEs. Gonadotropins (FSH, LH) and sex hormones were not measured in this case. However, the clinical utility of gonadotropin assessment in ICI‐induced hypophysitis is limited, particularly in the acute setting where the gonadal axis may be suppressed by critical illness, and sex steroid replacement is generally not indicated during acute illness [8]. Furthermore, in this postmenopausal patient with advanced malignancy and limited life expectancy, gonadotropin evaluation would not have altered management. Further studies are needed to establish more comprehensive guidelines for the monitoring and intervention of endocrine dysfunctions tied to ICI therapy. There is still no universally accepted diagnostic gold standard for ICI‐induced hypophysitis, especially in patients without classic MRI findings. The evolving diagnostic framework makes it challenging to confidently attribute all abnormalities to ICI‐induced hypophysitis versus overlapping conditions. Long‐term endocrine outcomes (e.g., persistence of adrenal insufficiency, need for lifelong hormone replacement) are not reported, limiting insights into the chronic disease burden associated with ICI‐induced hypophysitis. There is also a notable paucity of prospective data on optimal steroid dosing and long‐term outcomes [8]. Further research is needed to refine risk stratification and management algorithms.

This case focuses on early detection, systematic monitoring, and acute evidence‐based management of ICI‐induced hypophysitis. It also highlights the need for ongoing research to address gaps in diagnostic criteria, long‐term outcomes, and individualized management strategies. Long‐term endocrine outcomes (e.g., persistence of adrenal insufficiency, need for lifelong hormone replacement) are not reported, limiting insights into the chronic disease burden associated with ICI‐induced hypophysitis. Despite these limitations, this case highlights important clinical lessons regarding the early recognition and management of endocrine irAEs in patients receiving combination immune checkpoint inhibitor therapy.

## Author Contributions

**Nadia Grace Obaed:** conceptualization, data curation, formal analysis, investigation, writing – original draft, writing – review and editing. **Kadiyatu Fofana:** conceptualization, formal analysis, writing – original draft, writing – review and editing. **Ahmad Matarneh:** formal analysis, writing – original draft, writing – review and editing. **Sundus Sardar:** writing – review and editing. **Naman Trivedi:** writing – review and editing. **Nasrollah Ghahramani:** writing – review and editing.

## Funding

The authors have nothing to report.

## Consent

Signed informed consent was obtained directly from the patient's relatives or guardians.

## Conflicts of Interest

The authors declare no conflicts of interest.

## Data Availability

De‐identified data supporting the findings are available from the corresponding author on request.
